# Predicting Role of Interleukin‐33 in Determining the Development and Severity of Atopic Dermatitis

**DOI:** 10.1002/iid3.70351

**Published:** 2026-02-08

**Authors:** Ali‐reza Ghasemiyeh, Marzieh Heidarzadeh Arani, Fatemeh Riazian, Ali Aghajani, Mohammad Javad Azadchehr, Hossein Motedayyen

**Affiliations:** ^1^ Pediatric Department, School of Medicine Kashan University of Medical Sciences Kashan Iran; ^2^ Department of Biology, Science and Research Branch Islamic Azad University Tehran Iran; ^3^ Clinical Diagnostic Laboratory Matini Hospital, Kashan University of Medical Sciences Kashan Iran; ^4^ Infectious Diseases Research Center Kashan University of Medical Sciences Kashan Iran; ^5^ Autoimmune Diseases Research Center Kashan University of Medical Sciences Kashan Iran

**Keywords:** atopic dermatitis, disease severity, environmental allergens, food allergens, immunoglobulin E (IgE), interleukin‐33 (IL‐33)

## Abstract

**Introduction:**

Atopic dermatitis (AD), also referred to as atopic eczema, is one of the most common immunological disorders in children. Previous studies have suggested potential roles of interleukin‐33 (IL‐33) in the onset and progression of AD. This study investigated whether serum IL‐33 levels in children with AD are associated with disease severity, immunoglobulin E (IgE) sensitization to food or environmental allergens, and serum IgE concentration.

**Methods:**

The study included 62 children with newly diagnosed AD and 30 age‐matched healthy controls. Following an interview and confirmation of disease severity, 3mL of venous blood was collected from each participant. Sensitization to allergens was assessed using a skin prick test. Serum IL‐33 (ng/L) and IgE (IU/mL) levels were measured using enzyme‐linked immunosorbent assay (ELISA).

**Results:**

Among the 62 children with AD, 51 (82.3%) were sensitized to food allergens, whereas 11 (17.7%) were sensitized to environmental allergens. Most patients (75.8%) had mild AD. Serum IL‐33 and IgE levels were significantly elevated in patients compared with healthy controls. Both IL‐33 and IgE concentrations were significantly associated with moderate AD. However, IL‐33 levels were not correlated with age, gender, or allergen type. Receiver operating characteristic (ROC) analysis revealed that IgE levels demonstrated moderate diagnostic performance for AD (AUCc0.758), while IL‐33 showed weaker diagnostic value (AUC = 0.678). For predicting disease severity, IL‐33 exhibited strong performance, with the highest sensitivity (85.71%) and maximum specificity (70.21%) at a cut‐off of 331.32 ng/L (AUC = 0.862). In contrast, IgE was not a reliable predictor of AD severity.

**Conclusion:**

Serum IL‐33 levels are significantly correlated with AD severity and may serve as a predictive biomarker, whereas IgE shows limited utility for severity assessment. Further studies are warranted to validate the clinical applicability of IL‐33 in AD management.

## 1 Introduction

Atopic dermatitis (AD), also referred to as atopic eczema, is a prevalent chronic inflammatory disease with a global prevalence of 2%–20% in children and 2%–10% in adults, and its incidence is rising—especially in adolescents [1]. AD extremely impacts quality of life, causing anxiety, depression, sleep disturbance, and high socioeconomic costs [2]. AD typically manifests in early childhood, often within the first 6 months of life, although it can observe at any age [3]. It is characterized by erythematous, pruritic, and scaly lesions primarily affecting the face and extensor surfaces in infants, with a predilection for flexural areas such as the antecubital and popliteal fossae in older children and adults [1, 4, 5].

Although AD can be effectively managed with topical and/or systemic therapies, some patients exhibit disease that is refractory to conventional treatments, necessitating additional options such as immunosuppressive agents [6, 7]. Recently, several experimental and preclinical studies have reported novel therapeutic approaches and potential treatment targets for AD. For example, topical bosentan has demonstrated anti‐inflammatory impacts and reduction of disease severity in animal models of AD [8]. Similarly, other studies on mouse models of AD have indicated that bioactive compounds such as the phenolic fraction of *Salvia frigida* and the phytosterol fraction of *Chenopodium murale* have promising immunomodulatory effects and barrier‐restoring activities, in some cases comparable to or exceeding tacrolimus [9, 10]. These findings emphasize emerging directions in AD therapy, focusing on targeted modulation of immune pathways, and restoration of skin barrier function.

The pathogenesis of AD is multifactorial, involving intricate interactions between genetic predisposition, immune dysregulation, environmental factors, microbial dysbiosis, and skin barrier defects [1, 11, 12]. Genetic alterations in genes encoding epidermal barrier proteins, such as filaggrin, play a major role in barrier dysfunction, allowing increased penetration of allergens, microbes, and irritants [13]. Environmental exposures, including allergens, pollutants, microbial colonization, and climate, exacerbate the disease process by impairing barrier function and promoting inflammation [11].

Barrier disruption triggers the release of epithelial‐derived alarmin such as thymic stromal lymphopoietin (TSLP), interleukin‐25 (IL‐25), and interleukin‐33 (IL‐33) from keratinocytes, which drive Th2‐mediated inflammation [11, 14]. The activated Th2 cells secrete IL‐4, IL‐5, IL‐13, and the pruritogenic cytokine IL‐31, which promote immunoglobulin E (IgE) production, eosinophil activation, and intense itching [1, 11]. In chronic phases, other immune subsets (Th1, Th17, Th22) become involved, leading to increased IL‐22 and IL‐17 production, driving epidermal differentiation abnormalities mediated by S100 proteins [1].

Comorbidities of AD are diverse, encompassing atopic conditions such as asthma, allergic rhinitis, and food allergies—the “atopic march”—as well as non‐atopic associations including ocular disorders, psychiatric conditions (depression, anxiety), cardiovascular disease, autoimmune disorders, and increased malignancy risk [1, 2]. Notably, emerging evidence suggests a possible link between AD and other chronic inflammatory dermatoses, such as psoriasis, with shared and divergent immunological mechanisms—including overlapping Th17/Th22 pathways—potentially contributing to co‐occurrence or sequential development of these diseases [15, 16]. This finding emphasizes the systemic nature of AD‐associated immune dysregulation and underlines the importance of considering overlapping inflammatory pathways when evaluating comorbid skin disorders.

Interleukin‐33 (IL‐33) — a member of the IL‐1 family — is a key alarmin released by keratinocytes in response to allergens or *Staphylococcus aureus* toxins, suppresses FLG expression, enhances Th2 activation and cytokine production (IL‐4, IL‐5, and IL‐13), increases TSLP–OX40L signaling, thereby driving AD inflammation and barrier breakdown [17]. It also stimulates group 2 innate lymphoid cells (ILC2s) to produce type 2 cytokines, such as IL‐5 and IL‐13 [18]. This pleiotropic activity implicates IL‐33 in diverse biological processes, including inflammation, cancer, infectious diseases, tissue repair, and metabolic homeostasis, and diseases with immune pathophysiology [19].

In line with the roles of IL‐33 in allergic reactions, some studies pointing to an increased expression of IL‐33 in the keratinocytes of patients with AD [18]. Over‐expression of IL‐33, especially in keratinocytes, significantly contributes to the development of AD‐like eczema in IL‐33 transgenic mice [18]. *In vivo* studies have proposed that IL‐33 is sufficient for the development of AD by inducing IL‐31 production and thereby promote pruritus and scratching behavior [18]. Skin scratching enhances the release of IL‐33 from keratinocytes, which reduces the expression of filaggrin and claudin‐1. The reduced expressions of these proteins participate in reducing the skin barrier function. In turn, barrier destruction leads to the increased exposure of immune cells to allergens and enhanced release of IL‐33 [20, 21].

Given the central role of IL‐33 in amplifying immune responses and impairing barrier function, this study examined whether IL‐33 levels are associated with IgE sensitization to food and environmental allergens, total serum IgE concentrations, and their potential value in predicting AD diagnosis and disease severity.

## 2 Materials and Methods

### 2.1 Study Populations

The study included 62 children with newly diagnosed AD and 30 healthy controls without immune‐related disorders (Table 1). Participants were recruited from the allergy clinic of Shahid Beheshti Hospital, Kashan, Iran, from September 2023 and March 2024. AD and its severity were diagnosed by an allergy and immunology specialist and confirmed according to clinical features and guidelines in the management of AD [22, 23, 24]. Briefly, a diagnosis was confirmed when the clinical history and physical examination demonstrated an itchy skin condition (or parental report of scratching or rubbing in a child) plus three or more of the following criteria: 1) history of flexural involvement (including cheeks in children < 10 years old); 2) visible flexural eczema (including the forehead, cheeks, and outer limbs in children < 4 years old); 3) a personal history of asthma and hay fever; 4) a history of atopic disease in a first‐degree relative (for children < 4 years old); 5) history of generalized dry skin in the past year; and 6) onset of rash before the age of 2 years. Inclusion criteria were: 1) newly diagnosed AD; 2) age between 1 and 12 years; 3) no history of allergic and other inflammatory disorders influencing the production of IgE and IL‐33; 4) no treatment with drugs affecting immune system. Exclusion criteria included: 1) history of chronic inflammatory diseases; 2) presence of other immune‐related conditions affecting antibody or cytokine production. The study was approved by the Ethics Committee of Kashan University of Medical Sciences (IR. KAUMS. NUHEPM. REC.1401.074) and performed according to the declaration of Helsinki. The written informed consent was collected from legally authorized representatives of children prior to entering the study.

**Table 1:** Demographic, laboratory, and clinical characteristics of patients with AD.

	Conditions	Atopic dermatitis (*n* = 62)	Healthy subjects (*n* = 30)	*p* value
Gender	Male/female	32 (51.6%)/30 (48.4%)	10 (33.3%)/20 (66.7%)/	0.099 *
Age (mean ± SD)	Year	5.54 ± 3.50	6.00 ± 2.24	0.449 **
Allergen type	Food environmental	51 (82.3%)	—	—
		11 (17.7%)		
Disease severity	Mild	47 (75.8%)		
	Moderate	14 (22.6%)		
	Severe	1 (1.6%)		
Clinical characteristics	Pruritus/itching	14 (22.5%)	—	—
	Chronic relapsing eczema	32 (51.6%)		
	Family history of atopy	9 (14.51%)		
	Excoriation of skin	1(1.61%)		
	Dryness of skin	6 (6.67%)		
	Flexural lichenification	0 (0.0%)		
	Ichthyosis	5 (8.06%)		
	Recurrent conjunctivitis	2 (3.22%)		

* Chi‐squared test.

** Unpaired *t*‐test.

### 2.2 Diagnosis of Allergen Sensitization

Sensitization to food and environmental allergens was assessed using a skin prick test (SPT) or radioallergosorbent test (RAST). SPT was performed by an allergy and immunology specialist in an allergy clinic. Allergen extracts relevant to the Iranian population—tree nuts, peanut, wheat, soybean, cow's milk, fish, banana, rice, pepper, meat, sesame, potato, tomato, egg, house dust mite (HDM), Russian thistle pollen, and cat hair—were obtained from Stallergenes Greer (Antony Cedex, France). The test procedure followed standard techniques [25, 26]. Briefly, the skin of the anterior part of the forearm was cleaned with 70% alcohol. The volar forearm skin was cleaned with 70% alcohol, a drop of allergen extract was applied, and the skin was gently pricked. After 10–20 min, wheal diameter was measured; a reaction > 3mm was considered positive. Histamine chloride in saline served as the positive control, and isotonic NaCl as the negative control. RAST for allergen‐specific IgE was performed using a Pharmacia ImmunoCAP 250 analyzer (Phadia, Uppsala, Sweden) based on the manufacturer's protocols.

### 2.3 Assessment of Serum IL‐33 and IgE Concentrations

Serum IL‐33 level was quantified using a human IL‐33 enzyme‐linked immunosorbent assay (ELISA) kit (ZellBio GmbH, Ulm, Germany; Cat. No. ZB‐10044C‐H9648), with a detection range of 10–2000 ng/L and calibration standards of 0, 20, 100, 500, 1000, and 2000 ng/L. Total IgE was measured using the IgE AccuBind ELISA kit (Monobind Inc., Lake Forest, CA, USA; Cat. No. 2525‐300A), which had a detection range of 0–10000 IU/mL and calibration standards of 0, 5, 25, 50, 150, and 400 IU/mL. All measurements were performed in duplicate.

### 2.4 Statistical Analysis

Data were analyzed using GraphPad Prism 6 (GraphPad Software, USA). Results are presented as mean ± standard deviation (SD). The D'Agostino–Pearson test was applied to assess data normality. Normally distributed data were analyzed using unpaired *t*‐tests and one‐way analysis of variance (ANOVA), while non‐normally distributed data were analyzed using Mann–Whitney and Kruskal–Wallis tests. Spearman's correlation test was used for non‐normal data, and Chi‐square test was applied to examine categorical variables. Receiver operating characteristic (ROC) curve analysis was performed to determine the area under the curve (AUC), sensitivity, specificity, positive predictive value (PPV), and negative predictive value (NPV) of IL‐33 and IgE for diagnosing AD and assessing its severity. A *p*‐value < 0.05 was considered statistically significant.

## 3 Results

### 3.1 Patient Descriptions

Sixty‐two cases with AD and 30 healthy volunteers participated in the study (Table 1). The onset of clinical symptoms in subjects with AD varied and occurred from 1 year to 12 years of age. The age at which AD was confirmed varied from a range of 2 years old to 14 years (data not shown). As shown in Table 1, no significant difference was observed in the age and gender between patient and healthy groups. The most frequent food allergens associated with AD were cow's milk, egg, and peanut, while they were cat hair and HDM for environmental allergens. Of the 62 patients with AD, three cases had sensitivity to tomatoes, pepper, fish, bananas, and meat. Two cases suffered from sensitivity to rice, wheat, soybean, and flower pollen. Approximately, 75.8% of patients suffered from a mild form of AD (Table 1). The demographic and other information of volunteers are shown in Table 1. *The The association of IL‐33 and IgE with AD severity*

**Figure 1:**
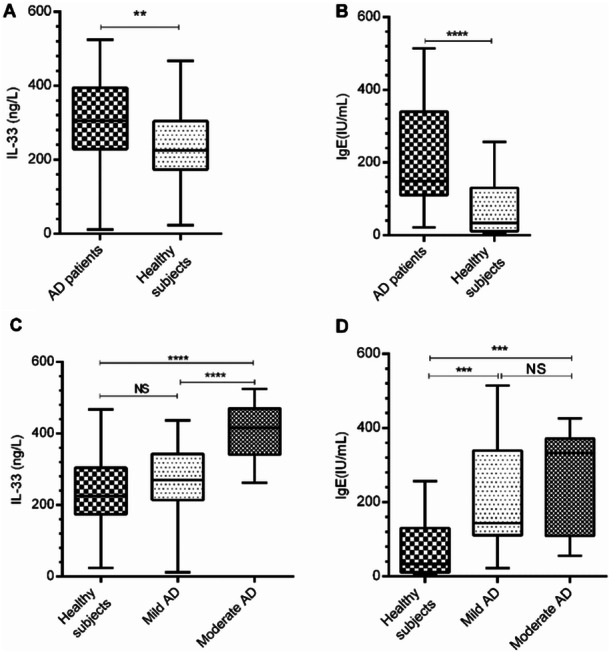
The serum concentrations of IL‐33 and IgE and their association with disease severity. (A and B) The serum levels of IL‐33 and IgE in patients with mild (*n* = 47), moderate (*n* = 14), and severe AD (*n* = 1) and healthy subjects (*n* = 30) were measured by ELISA. Data are the median and the quartile range. The groups were analyzed using Kruskal–Wallis and Mann–Whitney tests. ***p< 0.001, ****p< 0.0001.

To determine the relationship of serum IL‐33 and IgE with disease severity, the serum values of these immunological agents were assessed in participants. The results indicated that patients with AD had a significant increase in the level of IL‐33 compared to healthy subjects (Figure 1A, *p* < 0.01). The same trend was also observed in the serum IgE concentrations (Figure 1B, *p* < 0.001). Our data indicated that the values of IL‐33 and IgE were significantly associated with moderate AD (Figure 1C,D, *p* < 0.0001).

### 3.2 The Relationship of Serum IL‐33 Level With Serum IgE Concentration, Allergen Type, and Other Demographic Information

**Figure 2:**
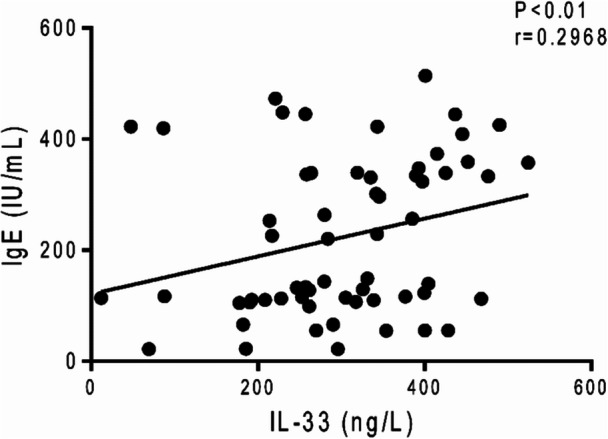
The relationship of serum IL‐33 level with serum IgE concentration. Spearman's rank correlation test was used to evaluate the relationship between serum IL‐33 and IgE concentrations in children with AD.

The correlation of serum IL‐33 level with serum IgE concentration, age, and gender of patients was evaluated. Serum IL‐33 level indicated a direct association with serum IgE concentration (Figure 2, *p* < 0.01, odds ratio (OR) = 0.2968; 95% CI = 0.04099 to 0.5161). Other results revealed that serum IL‐33 and IgE concentrations were not correlated to the age and gender of participants.

### 3.3 Predicting Roles of IL‐33 and IgE in AD Progression and Severity

**Figure 3:**
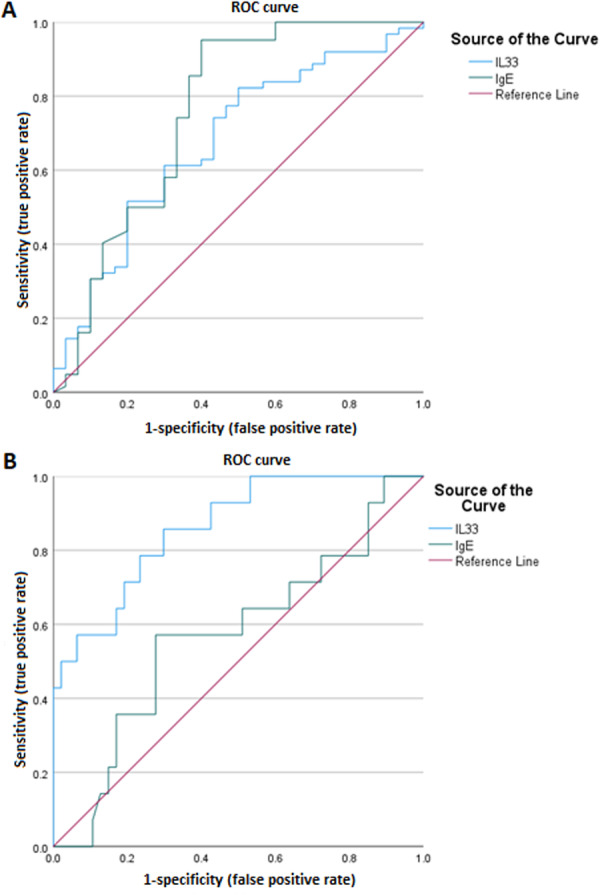
Diagnostic and predictive performance of serum IL‐33 and IgE in AD. (A) ROC analysis of IL‐33 and IgE for AD diagnosis. (B) ROC analysis of IL‐33 and IgE for predicting AD severity. Sensitivity and specificity were determined using a logistic regression model.

Using ROC AUC, we tried to determine the sensitivity and specificity of the serum levels of IL‐33 and IgE as diagnostic markers for AD. In this regard, serum IgE concentration had enough performance to diagnosis of AD (AUC ± SE [%95CI] = 0.758 ± 0.062 [0.658 to 0.842], Table 2 and Figure 3A), while serum IL‐33 level had a weak predictive role in AD diagnose (AUC ± SE [%95CI] = 0.678 ± 0.060 [0.572 to 0.772], Table 2 and Figure 3A). As diagnostic markers for severity of AD, the highest sensitivity (85.71%) and maximum specificity (70.21%) were found in the cut‐off point of IL‐33 serum level equal to 331.32 (AUC ± SE[%95CI] = 0.862 ± 0.054[0.749–0.937], Table 2 and Figure 3B). However, serum IgE concentration failed to be considered as a predictive biomarker in determining AD severity (AUC ± SE [%95CI] = 0.571 ± 0.091[0.437–0.697], Table 2 and Figure 3B).

**Table 2:** Sensitivity, specificity, and predicting values of IL‐33 and IgE in diagnosis and severity of AD.

Outcomes	Parameters	Cut point	AUC ± SE (%95CI)	Sensitivity (%)	Specificity (%)	PPV	NPV	−LR	+LR
Mild and moderate AD	IL‐33	211.67	0.678 ± 0.060 (0.572–0.772)	82.26	50	77.3	57.7	0.35	1.45
	IgE	38.11	0.758 ± 0.062 (0.658–0.842)	95.16	60	83.1	85.7	0.08	2.38
AD severity	IL‐33	331.32	0.862 ± 0.054 (0.749–0.937)	85.71	70.21	94.3	46.2	0.20	2.88
	IgE	—	0.571 ± 0.091 (0.437–0.697)	—	—	—	—	—	—

Abbreviations: AUC, area under curve; NPV, negative predictive value; PPV, positive predictive value.

## 4 Discussion

IL‐33 released by keratinocytes, endothelial cells, and immune cells in various tissues and organs plays a critical in both innate and adaptive immunity. In several organs, it appears to drive Th2 responses, implicating it in allergic and atopic diseases [20]. IL‐33 exerts its impacts through activation of the suppression of tumorigenicity 2 (ST2, IL‐1 receptor) on different cells, including mast cells and Th2 cells [20]. Therefore, the current study investigated whether a change in serum IL‐33 levels are correlated to AD severity and they can serve as a biomarker for predicting disease progression.

In the present study, the onset of clinical symptoms varied widely among patients, ranging from 1 to 12 years of age. Previous studies have demonstrated that the symptoms of AD typically occur before the age of 2 years; with approximately 60% of cases begining before 1 year of age. Kolb et al. reported that AD resolves by the age of 12 years in 60% of individuals [27]. while Spergel et al. found that 85% of cases begin in childhood and nearly 95% occur before the age of 5 years [28]. Similarly, other studies indicate that most cases manifest by 2 years of age [29]. The broader age range in our study may be partly explained by late referral and delayed diagnosis, likely due to mild disease forms and the use of home remedies. Importantly, in a cross‐sectional design, such delays between symptom onset and diagnosis could potentially influence serum IL‐33 levels, as prolonged disease duration and chronic inflammation may alter cytokine expression. Although our study did not record the symptom‐to‐diagnosis interval, future work should assess this variable to clarify whether disease chronicity alters IL‐33 levels and to improve the biomarker's clinical interpretability.

Having considered that certain allergens can induce allergic inflammation via proteolytic maturation of IL‐33, we also examined the relationship between serum IL‐33 levels and IgE sensitization to food or environmental allergens. Our data indicated that there was no significant association between IL‐33 and the type of allergen sensitization. Although this is the first report to find no such association, several studies have shown that allergens such as HDM, pollen, and fungi stimulate IL‐33 synthesis and release from keratinocytes through ATP‐mediated extracellular signaling [30, 31, 32]. Proteolytic allergens can rapidly cleave full‐length IL‐33 in its central ‘sensor’ domain, enhancing type 2 cytokine production by ILC2 [33]. In vivo studies have further demonstrated that IL‐33 signaling is essential for initiating Th2 responses to HDM and peanut allergens in allergic asthma and food allergy, potentially due to its strong capacity to induce OX40 ligand (OX40L) expression on dendritic cells and expand ILC2 [34]. These observations support a molecular mechanism for the rapid induction of allergic type 2 inflammation following allergen exposure, with important implications for the treatment of AD [34].

Although some reports have suggested that IL‐33 may have both pro‐ and anti‐inflammatory functions [35], the results of our study, along with others, support a predominantly pro‐inflammatory role. We observed significantly elevated serum IL‐33 levels in AD patients compared to healthy controls, accompanied by increased IgE levels. After adjusting for age and sex, IL‐33 levels were significantly higher in patients with moderate AD than in those with mild disease, whereas IgE levels did not differ significantly between these groups.

In addition to Tamagawa‐Mineoka et al. (2014), who reported elevated serum IL‐33 levels in adult AD patients and demonstrated a positive correlation with disease severity [36], other studies by Nygaard et al. (2016) [37] and Savinko et al. (2012) [21] indicated enhanced IL‐33 expression in both serum and lesional skin, further supporting its role in AD pathogenesis. However, most of these studies focused primarily on adult populations and chronic AD. Our work extends these findings by specifically examining children with newly diagnosed AD, a group less frequently characterized, and by assessing the predictive performance of IL‐33 for disease severity using ROC analysis. To our knowledge, this is one of the few pediatric studies to quantitatively evaluate IL‐33 as a potential biomarker for AD severity, contributing novel data on its diagnostic and prognostic potential in early disease stages.

Moreover, several recent clinical trials have investigated IL‐33 blockade as a therapeutic strategy in atopic diseases. Monoclonal antibodies targeting IL‐33 and its receptor ST2—such as etokimab (ANB020) and itepekimab (REGN3500)—have demonstrated reductions in Th2‐associated cytokines and clinical improvements in patients with moderate‐to‐severe asthma and AD [38, 39, 40]. Although results have been mixed, these therapeutic efforts highlight the translational relevance of IL‐33 as both a biomarker and a treatment target. Our findings further support this by indicating that serum IL‐33 reflects disease activity and may aid in identifying patients with more severe inflammation.

To evaluate the diagnostic and prognostic value of IL‐33 and IgE, we performed ROC curve analysis. An IL‐33 cut‐off point of 211.67 pg/mL (AUC = 0.678) and an IgE cut‐off point of 38.11 IU/mL (AUC = 0.758) showed the highest specificity and sensitivity for diagnosing AD. For assessing disease severity, an IL‐33 cut‐off point of 331.32 pg/mL (AUC = 0.862) provided the best diagnostic performance, while IgE levels lacked sufficient discriminatory power. The AUC values for IL‐33 and IgE in predicting disease severity were 0.862 and 0.571, respectively, suggesting that IL‐33, unlike IgE, can serve as a reliable biomarker for disease severity. Further studies with longitudinal designs are warranted to confirm these findings and to evaluate the influence of disease chronicity and timing of diagnosis on IL‐33 expression.

## 5 Conclusion

The results of the present study, together with previous reports, provide further evidence for the potential role of IL‐33 in Th2‐mediated inflammation in AD. We observed a significant association between elevated serum IL‐33 and IgE levels, and our data suggest that IL‐33 levels may correlate with AD severity independently of age and sex. Moreover, serum IL‐33 demonstrated good discriminative ability for assessing disease severity, although its diagnostic value for detecting AD was limited. Importantly, this work expands upon previous studies by analyzing pediatric patients with newly diagnosed AD and by evaluating IL‐33 as a quantitative biomarker using ROC‐based thresholds. Several limitations should be acknowledged. First, the absence of a comparison group with other chronic inflammatory dermatoses (e.g., chronic eczema, psoriasis) limits our ability to determine whether the observed IL‐33/IgE association is specific to AD or reflects a broader inflammatory response. Second, this was a single‐center study, which may restrict the generalizability of our findings to more diverse populations. Third, the interval between symptom onset and diagnosis was not recorded, yet in a cross‐sectional design, delayed presentation and prolonged disease duration could influence cytokine expression, including IL‐33. Fourth, IL‐33 levels were measured solely by ELISA without validation using complementary methods such as quantitative PCR or Western blotting; future studies incorporating such approaches would help confirm these results at both the protein and mRNA levels. Finally, the lack of repeated IL‐33 measurements over time precludes assessment of its dynamic role in disease progression or treatment response.

Despite these constraints, our findings provide a foundation for future research. Multicenter longitudinal studies with larger sample sizes, inclusion of control groups with other chronic dermatoses, documentation of symptom‐to‐diagnosis intervals, and incorporation of multiple molecular validation methods are warranted. Such studies could clarify whether IL‐33 is a disease‐specific driver or a general inflammatory biomarker and better define its clinical utility in AD.

## Author Contributions

**Ali‐reza Ghasemiyeh:** investigation, writing – original draft. **Marzieh Heidarzadeh Arani:** methodology, resources. **Fatemeh Riazian:** conceptualization, investigation. **Ali Aghajani:** investigation, methodology. **Mohammad Javad Azadchehr:** data curation, formal analysis. **Hossein Motedayyen:** supervision, writing – review and editing.

## Conflicts of Interest

The authors declare no conflicts of interest.

## Data Availability

The original contributions presented in the study are included in the article. Further inquiries can be directed to the corresponding authors.
